# Associations between digital health literacy and health system navigating abilities among Norwegian adolescents: validating the HLS_19_-DIGI scale using Rasch modeling

**DOI:** 10.1186/s12889-024-19405-w

**Published:** 2024-07-30

**Authors:** Christopher Le, Øystein Guttersrud, Diane Levin-Zamir, Robert Griebler, Hanne Søberg Finbråten

**Affiliations:** 1 Department of Health and Nursing Sciences, Faculty of Social and Health Sciences, Inland Norway University of Applied Sciences, PO Box 400, Elverum, N-2418 Norway; 2 Norwegian Centre for Science Education, Faculty of Mathematics and Natural Sciences, University of Oslo, PO Box 1106, Blindern, Oslo, N-0317 Norway; 3 School of Public Health, University of Haifa; Department of Health Education and Promotion, Clalit Health Services, 101 Arlozovov St, Tel Aviv, Israel; 4 Competence Centre Health Promotion and Healthcare, Austrian National Public Health Institute, Vienna, 1010 Austria

**Keywords:** Adolescent, Digital health literacy, Health system navigating abilities, HLS_19_-DIGI, Rasch modeling

## Abstract

**Background:**

Despite increasing global attention to health literacy and adolescents’ digital health information seeking, no unidimensional instruments measuring digital health literacy (DHL) in adolescents have reportedly been validated using Rasch modeling. Moreover, the evidence of adolescents’ abilities to navigate the health system (NAV-HL) in light of their DHL proficiency is still scarce. Therefore, our study aims to evaluate the psychometric properties of a DHL instrument (HLS_19_-DIGI scale) in order to investigate DHL in adolescents and young adults aged 16–25 and associations with abilities to navigate the health system.

**Methods:**

A population-based cross-sectional survey among 890 Norwegian adolescents was conducted during April–October 2020 using computer-assisted telephone interviewing. Rasch modeling, independent samples t-test, chi-square test, and binary regression models were used to analyze the data.

**Results:**

The HLS_19_-DIGI scale was sufficiently unidimensional, whereas no differential item functioning or disordered response categories were observed. However, relatively poor targeting was revealed indicating too many easy items for the target population. Yet, a high proportion (54%) of low DHL proficiency in adolescents was observed, as well as DHL was positively associated with the abilities to navigate the health system.

**Conclusions:**

The HLS_19_-DIGI scale is considered a sufficiently unidimensional and valid instrument for measuring DHL in adolescents, which may be a useful tool for health authorities, public health workers, and health service providers. While DHL affects adolescents’ abilities to navigate the health system, future research should measure and examine their ability to utilize digital health services, separately.

**Supplementary Information:**

The online version contains supplementary material available at 10.1186/s12889-024-19405-w.

## Introduction

In several Western countries, adolescents are increasingly expected to take responsibility for their own health [1]. They are exposed to a wealth of health information [2], while research has suggested that their general health literacy (GHL) in terms of their abilities to access, understand, critically appraise, and apply such information may be insufficient [3]. Correspondingly, a conceptualization of access to healthcare by Levesque et al. [4] refers to five relevant abilities of the population: (1) to perceive, (2) to seek, (3) to reach, (4) to pay, and (5) to engage. These abilities largely reflect the health system navigating skills (item content) in the Navigation Health Literacy (NAV-HL) scale of the WHO Action Network on Measuring Population and Organizational Health Literacy (M-POHL) [5–7]. Furthermore, ever-increasing digital transformation in healthcare should also warrant policy-makers, practitioners, and researchers to acknowledge the importance of understanding people’s capabilities in using these resources and technologies for maintaining and/or promoting their health.

In addition to GHL and NAV-HL, digital skills are needed due to continuing digitization of health information and the healthcare services. These skills, called digital health literacy (DHL), are defined by the HLS_19_-Consortium of M-POHL as “*the ability to search for*,* access*,* understand*,* appraise*,* and apply online health information*,* the ability to formulate and express questions*,* opinion*,* thoughts*,* or feelings when using digital devices*” [6, p.278]. While knowing that the terms DHL and eHealth literacy might be used interchangeably in research, we prefer the term DHL in accordance with the HLS_19_-Consortium of M-POHL [6, p. 278]. Specifically, the term *digital health* is more inclusive by means of their close relationship to mHealth (mobile) and artificial intelligence, and other emerging areas of innovation and information technology. DHL, including media health literacy and eHealth literacy throughout the lifespan have been explored in a number of studies; in childhood and adolescence [7–9] and during adulthood included the elderly [10, 11]. Moreover, it has also been studied from the aspect of cultural transition [12], health conditions [13], and health behaviors [14]. While research suggest that there is a correlation between GHL and DHL [8, 9], it is also acknowledged that quality assured digital health information can be used to promote healthy lifestyles and help prevent physical and mental illness over time [10–12]. Such information services can be part of a broad-based public health program commencing early in the life course and continuing throughout the lifespan. For instance, through school, adolescents are well served by the Internet. However, such information is only useful for those who are capable to seek, understand, appraise, and apply the accessed information effectively [13]. Acknowledging that these abilities among adolescents may be insufficient [3], mainstreaming access to such information, therefore, can enable young users to better understand their own or relatives’ health and illnesses, and thereby empower patients in their interactions with health professionals. Thus, people who are capable of using digital health information for making decisions about their own health are expected to be more resourceful from a life course perspective. Accordingly, this is what we refer to as a component of DHL that involves the skills required for using search engines, mastering search strategies and critically appraising sources and identifying relevant and valid digital health information [5].

During adolescence, people are more likely to develop addictive behavior [14] and mental illness [15–17] as well as they are less likely to use health promoting and disease preventing services comparing to adults [18, 19]. Among young people, research revealed also a meaningful relationship between GHL and health behavior [20] as well as various health outcomes [21, 22]. Nonetheless, empowering adolescents to deal with health information requires sufficient GHL proficiency [23], and the same inevitably applies to DHL in a digitized era of health information and the health system [24, 25]. DHL is also referred to as the individual, social, and technical competencies that are essential for digitally searching, finding, understanding, and using health information [6, 26]. This has a number of potential consequences for individuals’ health in the broadest sense, as behavior in the digital spaces requires particular skills, in terms of digital health literacy, to be able to promote and maintain health and wellbeing, in addition to prevent or deal with illnesses.

Recently, a scoping review to identify available tools to measure DHL [15], which was built on the concurrently existing research [27], revealed that eHEALS [28] was the most commonly used measure of DHL. Notably, the eHEALS measure was originally developed and validated among adolescents aged 13–21. However, the eHEALS was mostly identified as a two-factor scale when applied to young people [29]. Summing up scores of individual items into a total score of DHL requires a unidimensional scale [30]. Further, Faux-Nightingale et al. [31] suggested that eHEALS does not provide any fixed assessment or score to indicate, for instance, people’s ability to interact with digital health-related resources.

Accordingly, the HLS_19_-Consortium of M-POHL developed a parsimonious unidimensional HLS_19_-DIGI scale for measuring people’s DHL [6], hereafter called HLS_19_-DIGI. The development was conceptually based on the comprehensive concept and definition of GHL but is expanded to include the characteristics of digital information sources [6]. In this study, we provisionally refer to this instrument as a measure for DHL among adult populations as it had not been validated for use among adolescents.

To our knowledge, no other unidimensional DHL instruments have been properly validated in adolescents aged 16 years and over [31]. In addition, in the Health Literacy Tool Shed database we did not succeed in finding any instruments specifically for DHL, that were validated for use among adolescents [32]. Meanwhile, eHEALS is another commonly used eHealth literacy instrument [28]. As it was mostly identified as a two-factor scale with no fixed score to indicate a proficiency [31], its applicability has weakened [29, 31]. Consequently, our study aimed to investigate digital health literacy in young people using the newly developed instrument HLS_19_-DIGI and explore its association with health system navigating abilities. This was operationalized by (1) evaluating the psychometric properties of the HLS_19_-DIGI applied in adolescents and young adults aged 16–25 years; (2) describing the distribution of DHL proficiency for the target group; and finally, (3) exploring the associations between DHL and adolescents’ NAV-HL.

## Methods

### Sampling and data collection

This study used data from the Norwegian part of Health Literacy Survey 2019–2021 (HLS_19_) [5], that was collected using computer assisted telephone interviews during April–October 2020. The data collection was carried out in two waves and stratified by age groups (x8), genders (x2), and counties (x11), of which the data used in this study included 2 out of 8 age groups (16–17 and 18–25). A detailed description of the data collection can be found in Le et al. [33]. Out of 6000 participants, 890 participants met our inclusion criteria “adolescents aged 16–25”. Notwithstanding, the NAV-HL data (*n* = 471) was collected only in the second wave of the data collection. In Norway, there are health clinics for adolescents aged 13–20, but many clinics treat people even up to 25 years old. In this study, we therefore labeled the sampled population as “adolescents” and may interchangeably refer to the terms “young people” and “young adults”.

### Measures

The HLS_19_-DIGI instrument that measures DHL, consists of eight items measuring the ability to search for, access, understand, appraise, and apply online health information, for instance, “how easy or difficult is it to use the proper words or search query to find the information you are looking for?”, or “…to judge whether the information is applicable to you?”. The instrument uses a 4-point rating scale with the response categories; (1) very difficult, (2) difficult, (3) easy, and (4) very easy. A “don’t know” response category was used when stated spontaneously by the participants, which was recoded to missing data in the analyses.

In combination with the HLS_19_-DIGI instrument, we also collected data on NAV-HL (full text/description in Table 1) based on the Norwegian version [5] of the HLS_19_-NAV scale of M-POHL [7], and sociodemographic factors such as age, gender, education, self-perceived social status, and self-reported financial deprivation. All variables were dichotomized in the analyses except for DHL proficiency.

**Table 1:** Digital health literacy (in logits) using the HLS_19_-DIGI by sociodemographic characteristics and health system navigating abilities (NAV-HL)

	*n* (%) /mean (sd)^1^	DHL mean (sd)	*P* value
**Sociodemographic characteristics**			
**Age (** *n* ** = 890)**			
mean (sd)	21 (2.9)		
median	21		
16-20yo	436 (49.0)	1.271 (1.45)	**0.002**
21-25yo	454 (51.0)	1.621 (1.72)	
**Gender (** ***n*** ** = 890)**			
male	459 (51.6)	1.561 (1.60)	0.059
female	431 (48.4)	1.348 (1.60)	
**Education (** ***n*** ** = 890)**			
education years, mean (sd)	13 (2.3)		
below and equal to upper secondary education	684 (76.9)	1.409 (1.57)	0.137
above upper secondary education	201 (22.6)	1.608 (1.71)	
missing	5 (0.5)		
**Self-perceived social status (** ***n*** ** = 890)**			
mean (sd)	6 (1.6)		
lower (1–5)	245 (27.5)	1.291 (1.64)	0.061
higher (6–10)	591 (66.4)	1.532 (1.59)	
missing	54 (6.1)		
**Financial deprivation** ^**2**^ **(** ***n*** ** = 890)**			
no	704 (79.1)	1.525 (1.61)	**< 0.001**
yes	90 (10.1)	0.862 (1.43)	
missing	96 (10.8)		
***Health system navigating abilities (NAV-HL)***			
**NAV1 (** ***n*** **= 471)** “*…to understand information on how the health care system works?*”			
very easy and easy category	294 (62.4)	2.029 (1.53)	**< 0.001**
very difficult and difficult category	151 (32.1)	0.866 (1.42)	
missing	26 (5.5)		
**NAV2 (** ***n*** **= 471)** “*…to judge which type of health service you need in case of a health problem?*”			
very easy and easy category	361 (76.6)	1.878 (1.54)	**< 0.001**
very difficult and difficult category	96 (20.4)	0.737 (1.46)	
missing	14 (3.0)		
**NAV3 (** ***n*** **= 471)** “*…to judge to what extent a health insurance covers your need of a particular health service?*”			
very easy and easy category	212 (45.0)	2.229 (1.56)	**< 0.001**
very difficult and difficult category	217 (46.1)	1.037 (1.44)	
missing	42 (8.9)		
**NAV4 (** ***n*** **= 471)** “*…to find out if a particular healthcare service requires a deductible?*”			
very easy and easy category	262 (55.6)	1.965 (1.58)	**< 0.001**
very difficult and difficult category	182 (38.6)	1.213 (1.50)	
missing	27 (5.8)		
**NAV5 (** ***n*** **= 471)** “*…to understand information on ongoing health care reforms that might affect your health care?*”			
very easy and easy category	223 (47.3)	2.054 (1.58)	**< 0.001**
very difficult and difficult category	208 (44.2)	1.205 (1.58)	
missing	40 (8.5)		
**NAV6 (** ***n*** **= 471)** “*…to find out about your rights as a patient or user of the health care system?*”			
very easy and easy category	269 (57.1)	2.047 (1.54)	**< 0.001**
very difficult and difficult category	182 (38.6)	1.042 (1.48)	
missing	20 (4.3)		
**NAV7 (** ***n*** **= 471)** “*…to decide for a particular health service if you need it?*”			
very easy and easy category	325 (69.0)	1.903 (1.56)	**< 0.001**
very difficult and difficult category	121 (25.7)	0.978 (1.42)	
missing	25 (5.3)		
**NAV8 (** ***n*** **= 471)** “*…to find information on the quality of a particular health service?*”			
very easy and easy category	258 (54.8)	1.933 (1.59)	**< 0.001**
very difficult and difficult category	184 (39.1)	1.251 (1.53)	
missing	29 (6.1)		
**NAV9 (** ***n*** **= 471)** “*…to judge if a particular health service covers your healthcare need?*”			
very easy and easy category	325 (69.0)	1.935 (1.52)	**< 0.001**
very difficult and difficult category	123 (26.1)	0.893 (1.52)	
missing	23 (4.9)		
**NAV10 (** ***n*** **= 471)** “*…to know how to get an appointment in the primary healthcare service?*”			
very easy and easy category	416 (88.3)	1.722 (1.59)	**< 0.001**
very difficult and difficult category	43 (9.2)	0.975 (1.33)	
missing	12 (2.5)		
**NAV11 (** ***n*** **= 471)** “*…to find out how user organizations or NGOs may help you to orientate yourself in the health care system?*”			
very easy and easy category	287 (60.9)	1.922 (1.62)	**< 0.001**
very difficult and difficult category	123 (26.1)	1.107 (1.41)	
missing	61 (13.0)		
**NAV12 (** ***n*** **= 471)** “*…to locate the right contact person for your need within a health care institution?*”			
very easy and easy category	318 (67.5)	1.934 (1.58)	**< 0.001**
very difficult and difficult category	121 (25.7)	0.950 (1.45)	
missing	32 (6.8)		

*Note* DHL: Standardized score (Person location estimates in logits) of digital health literacy (DHL) by means of the HLS_19_-DIGI. Higher values indicate higher DHL

^1^ categorical data: frequencies, n (percentage [%]); continuous data: mean (standard deviation [sd])

^2^ How easy or difficult is it for you to pay all bills at the end of month?

Self-perceived social status was measured using: [“On the following scale, step ‘1’ corresponds to “the lowest level in the society”; step ‘10’ corresponds to “the highest level in the society”. Could you tell me, how would you rank yourself?”], and self-reported financial deprivation was measured using: [“On a scale from “very easy” to “very difficult”, how easy is it for you to pay all bills at the end of the month?”]. The latter measure may be understood as “relative” as the youngest age group may be more dependent on the socioeconomic status of their parents, which would indirectly affect the 16 year-old adolescent’s ability to “pay the bills at the end of the month”. Therefore, we believe this factor was as relevant to the 16 year-old as it was to the older participants.

### Data analysis

#### Rasch modeling

Among different versions of Rasch model, we applied the partial credit model (PCM) [34] due to polytomously scored items in the HLS_19_-DIGI scale [35]. Using Rasch modeling, we evaluated data-model fit [33], dimensionality [30, 36, 37], targeting [38], reliability [39–41], item fit [42–46], differential item functioning (DIF) [47], and the ordering of response categories [47, 48]. Full description of the Rasch-procedure is available in Le et al. [33] and in the Supplementary text file. Along with the Rasch procedure outlined in the Supplementary text file, response dependency is indicated by residual correlations above 0.3 [49, 50].

#### Other statistical analyses

Independent samples t-test was used to compare the mean score of HLS_19_-DIGI between two independent groups, while the chi-square test was used to explore the differences of DHL (levels) across several sociodemographic factors (binary/categorical variables). Binary logistic regression models with dichotomized NAV-HL items as the dependent variables and DHL proficiency (raw score being transformed into person-location estimates in terms of logit values) as the independent variable were conducted. We also applied the “Wright’s method” as set forth in Guttersrud et al. [51] to estimate the levels of DHL proficiency. Statistical significance was set at 5% level.

## Results

### Characteristics of the participants

The study included 890 participants with a slight predominance of males (Table 1). Due to different waves of data collection [33], a smaller sample (*n* = 471) was applied to the variables concerning NAV-HL. Almost 80% of the participants have an education level at or below upper secondary school. Two-thirds report belonging to upper social level and above three out of four report no economic deprivation.

### Overall data-model fit and unidimensionality of the HLS_19_-DIGI scale

**Fig. 1:**
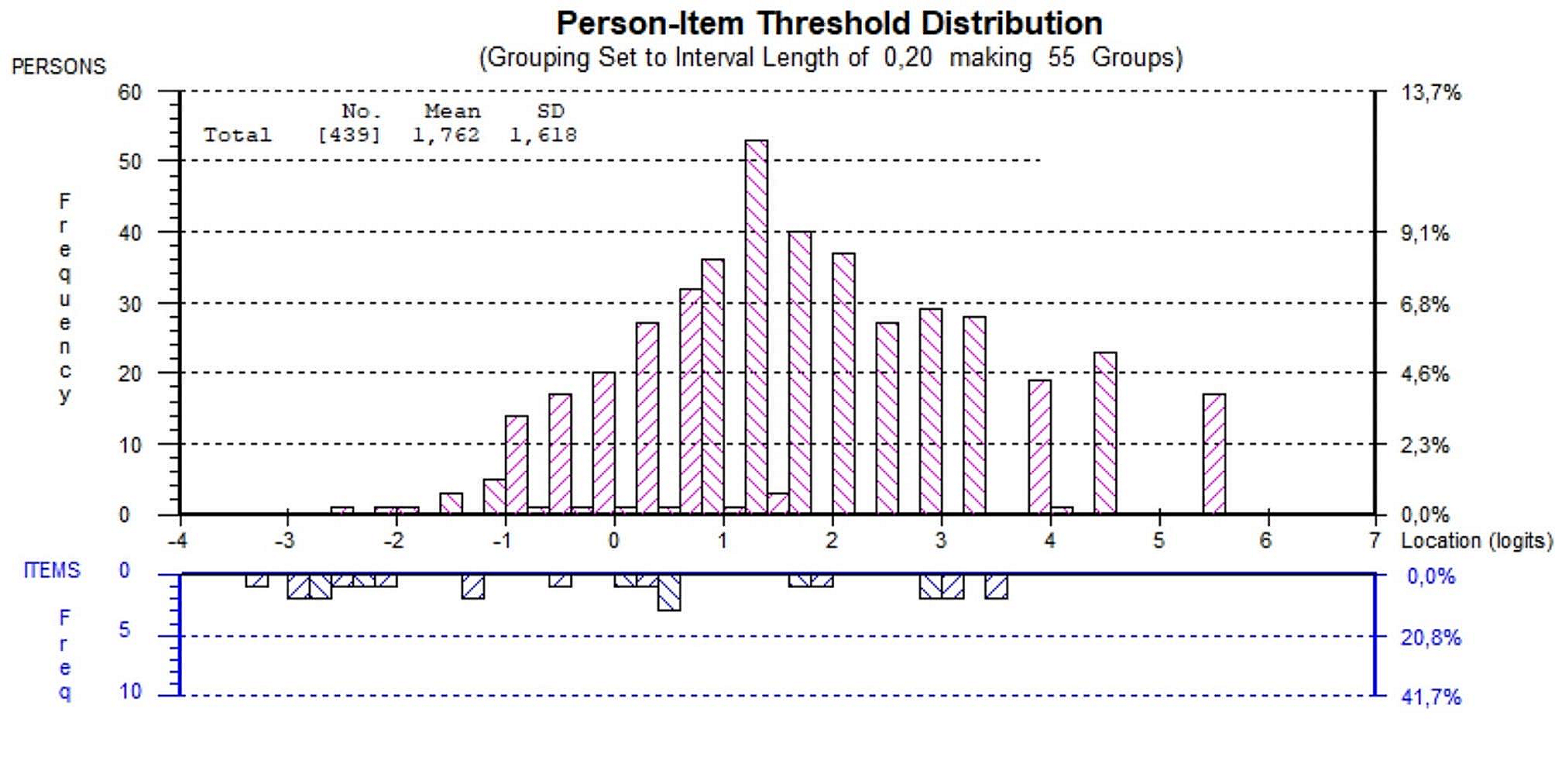
Targeting of the HLS_19_-DIGI scale

The HLS_19_-DIGI scale could be considered sufficiently unidimensional as the proportion of individuals with significantly different person-location estimates on the compared subscales is slightly above 5% (Table 2). Although the reliability indexes (PSR, PSI, and Omega) displayed good internal consistency, the scale could be better targeted to the population applied, as the distribution of person locations were right -skewed compared to the item-threshold locations. This indicated that the items are perceived as quite easy relative to the target population (Fig. 1), showing a ceiling effect.

**Table 2:** Unidimensionality, overall data-model fit, and reliability by applying Rasch modeling of the HLS_19_-DIGI

	HLS_19_-DIGI
	HLS_19_ Consortium 2021
**Unidimensionality t-tests (CI)** ^**RUMM**^	
number significant tests	33
out of:	422
dim(%)	7.82%
proportion lower 95% CI	5.7%
**Chi-square interaction** ^**RUMM**^	
total item chi-square	58.27
df	32
probability	0.003
**Mean (SD) in logits** ^**RUMM**^	
item fit residual	-1.33 (1.68)
person fit residual	-0.43 (1.25)
**Mean person location in logits** ^**RUMM**^	1.762
**Reliability**	
Omega (by Excel-based tool)^Mplus^	0.95
PSI based on PMLE^RUMM^	0.82
PSR (MMLE/WLE)^CQ^	0.82/0.82
**Log-likelihoods** ^**CQ**^	
Deviance (ep)	6,211 (25)
AIC (ep)	6,261 (25)

Note * total item chi-square is significant at 5%-level indicating significant deviation between the observed data and what was expected from the Rasch model; dim(%): proportion of individuals with significantly different person-location estimates (below 5% confirms unidimensionality); proportion lower 95% CI: lower than 5% confirms acceptable unidimensionality; df: degree of freedom; SD: Standard deviation; SE: Standard error; Omega: internal consistency reliability; PSI: person separation index; PSR: person separation reliability; PMLE: pairwise maximum likelihood estimate; MMLE: marginal maximum likelihood estimate; WLE: Warm’s mean likelihood estimate; Deviance: deviance statistics; ep: total number of estimated parameters; AIC: Akaike Information Criterion; RUMM: RUMM2030 software; CQ/ConQuest: ConQuest 5 software

The overall chi-square statistic was significant indicating problems at the individual item level (Table 2). In turn, this could be seen in relation to the t-value above 1.96 (item5: t-value = 2.3) in Table 3 that indicates a poorly fitting item in term of under-discrimination relative to the Rasch model. However, a smaller sample size that at random was gradually reduced to *n* = 305 and below displayed non-significant overall chi-square statistics.

**Table 3:** Item characteristics, disordered response categories, and DIF of the HLS_19_-DHL

CD	Item no.	Item: On a scale from very difficult to very easy, how easy would you say it is:	1-dimensional analysis HLS_19_- DHL							
			ConQuest				RUMM			
			Infit^w^ MNSQ	CI		T- value	Item estimate	SE	Ordered	DIF*
				**lb**	**ub**					
F	1	to use the proper words or search query to find the information you are looking for?	0.99	0.86	1.14	-0.1	-0.678	0.094	yes	none
U	2	to find the exact information you are searching for?	1.13	0.87	1.13	1.9	0.177	0.086	yes	none
J	3	to understand the information?	0.95	0.86	1.14	-0.8	0.113	0.087	yes	none
A	4	to judge whether the information is reliable?	0.96	0.87	1.13	-0.6	0.542	0.086	yes	none
F	5	to judge whether the information is offered with commercial interests?	**1.16**	0.87	1.13	**2.3**	0.098	0.086	yes	none
U	6	to visit different websites to check whether they provide similar information about the topic?	1.04	0.86	1.14	0.6	-0.884	0.094	yes	none
J	7	to judge whether the information is applicable to you?	0.91	0.87	1.13	-1.4	0.393	0.084	yes	none
A	8	to use the information to help solve a health problem?	0.93	0.87	1.13	-1.2	0.240	0.090	yes	none

*Note *^w^weighted fit MNSQ, one-dimensional model using ConQuest 5; ^u^A t-value > 1.96 indicates a poorly fitting item in terms of under-discrimination relative to the Rasch model

DIF: differential item functioning; *Bonferroni-adjusted 5% has been used to assist detecting possible significant deviations due to DIF; Ordered: “yes” refers to an item with none disordered response categories

F = Find; U = Understand; J = Judge; A = Apply; lb: lower bound; ub: upper bound; CI = confidence interval; T-value = similar to the z standardized fit statistics in unidimensional Rasch analyses; MNSQ = mean square value; SE = Standard Error

### Rasch analyses at item level for the HLS_19_-DIGI

Applying unidimensional Rasch modeling all items had acceptable infit values (Table 3) except for item 5, which had a T-value of 2.3 meaning that the item under-discriminated relative to the PCM. No disordered response categories were observed, neither items displaying differential item functioning (Table 3). Moreover, no residual correlations above 0.3 were observed, which indicates none-significant response dependence between any two items (Table 4).

**Table 4:** Residual correlations and response dependence between any two items of the HLS_19_-DIGI

Item	I0048	I0049	I0050	I0051	I0052	I0053	I0054	I0055
I0048								
I0049	0,138							
I0050	-0,015	-0,036						
I0051	-0,243	-0,276	-0,148					
I0052	-0,198	-0,281	-0,256	0,05				
I0053	-0,195	-0,299	-0,188	-0,113	0,044			
I0054	-0,225	-0,255	-0,085	-0,145	-0,282	-0,04		
I0055	-0,211	-0,142	-0,19	-0,15	-0,224	-0,112	0,121	

#### Levels of digital health literacy proficiency

Applying the Wright method, three distinctive levels (1–3) of DHL were found starting from a cut-point of 19, 24, and 29 out of 32 (Table 5), with level 1 labeled as the lowest and level 3 as the highest, respectively.

**Table 5:** Proportion of participants at three statistically distinct digital health literacy levels (HLS_19_-DIGI cut-points) across person factors

Health literacy level (cut-points)	Total sample *n* (%)	Gender *n* (%)		Age *n* (%)		Education *n* (%)		Financial deprivation *n* (%)		Self-perceived social status *n* (%)	
		Male	Female	16–20	21–25	Below^1^	Above^2^	Yes	No	Lower^3^	Higher^4^
		*p-value* = 0.152		*p-value* = **0.001**		*p-value* = 0.506		*p-value* = **0.018**		*p-value* = **0.041**	
Below Level 1 (5–18)	101 (12)	45 (11)	56 (14)	51 (13)	50 (12)	78 (13)	23 (12)	74 (11)	16 (20)	39 (18)	56 (10)
Level 1 (19–23)	336 (42)	155 (39)	181 (44)	177 (46)	159 (38)	263 (43)	69 (37)	260 (40)	39 (48)	86 (39)	226 (42)
Level 2 (24–28)	277 (34)	150 (38)	127 (31)	131 (34)	146 (34)	206 (33)	70 (38)	231 (36)	22 (27)	73 (33)	190 (35)
Level 3 (29–32)	95 (12)	50 (12)	45 (11)	28 (7)	67 (16)	70 (11)	25 (13)	80 (13)	4 (5)	22 (10)	69 (13)
n	809	400	409	387	422	617	187	645	81	220	541

Note: ^1^ Upper secondary school or below; ^2^ Above secondary school; ^3^ Level 1–5; ^4^ Level 6–10

While 46% are at or above level 2, more than half of the respondents (54%) are at or below level 1. People who achieved level 1 would typically perceive it as “easy” to *use the proper words or search query to find the information they are looking for* (item1) and to *visit different websites to check whether they provide similar information about the topic* (item6). Based on the content of these items, we can generalize that people who scored 19 or above probably can use digital resources to access quality assured information. People who achieved level 2 (sum score of 24 or above) would typically perceive the rest of the items as “easy”, which are item2 [*to find the exact information you are searching for*], item3 [*to understand the information*], item4 [*to judge whether the information is reliable*], item5 [*to judge whether the information is offered with commercial interests*], item7 [*to judge whether the information is applicable to you*], and item8 [*to use the information to help solve a health problem*]. This may be generalized that people who gained a score of 24 or above probably are able to understand, appraise, and apply the digital information accessed.

### Abilities to navigate the healthcare system by levels of digital health literacy

**Fig. 2:**
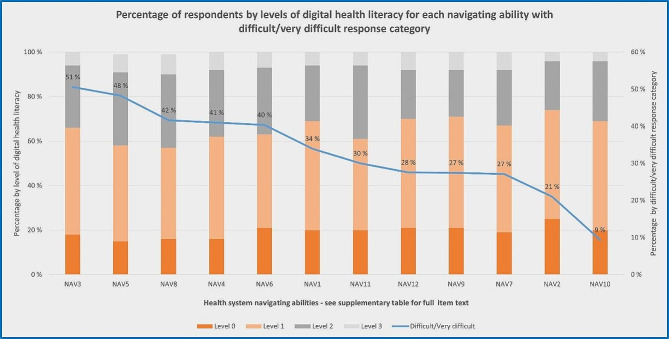
Abilities to navigate the healthcare system in light of levels of digital health literacy. *Note*: Percentage of difficult/very difficult without missing

More than 38% (40% excluding missing) of the participants report (very) difficult on NAV3, NAV4, NAV5, NAV6, and NAV8 (Table 1). Adjusted for age, gender, education, self-perceived social status, and self-reported financial deprivation, the results in Table 6 show that DHL is associated with all these NAV-HL items (*b* ranged from 0.07 to 0.11, *p* < .001). We also observed that more than 50% (ranged 57–74%) of the target population who experienced health system navigating abilities as (very) difficult are located at or below level 1 of DHL proficiency (Fig. 2).

**Table 6:** Binary logistic regression with NAV-abilities by DHL and covariates

DHL (logits)	NAV3		NAV4		NAV5		NAV6		NAV8	
	Coef. (OR)	*p*-value	Coef. (OR)	*p*-value	Coef. (OR)	*p*-value	Coef. (OR)	*p*-value	Coef. (OR)	*p*-value
(unadjusted)	0.53 (1.69)	< 0.001	0.32 (1.38)	< 0.001	0.36 (1.44)	< 0.001	0.45 (1.57)	< 0.001	0.28 (1.33)	< 0.001
DHL (adjusted)	0.54 (1.71)	**< 0.001**	0.31 (1.36)	**< 0.001**	0.39 (1.48)	**< 0.001**	0.47 (1.61)	**< 0.001**	0.29 (1.34)	**< 0.001**
Age	0.01 (1.01)	0.897	0.11 (1.12)	**0.024**	− 0.06 (0.95)	0.264	− 0.01(1.00)	0.924	− 0.07 (0.93)	0.177
Gender (female)	− 0.37 (0.69)	0.120	− 0.20 (0.82)	0.381	− 0.05 (0.95)	0.826	− 0.15 (0.86)	0.517	− 0.29 (0.75)	0.194
Education (above)	− 0.45 (0.64)	0.169	− 0.84 (0.43)	**0.006**	− 0.41 (0.67)	0.186	− 0.93 (0.39)	**0.003**	− 0.50 (0.61)	0.095
Social status (higher)	0.66 (1.93)	**0.015**	0.01 (1.01)	0.964	0.30 (1.35)	0.239	0.22 (1.24)	0.398	− 0.24 (0.78)	0.340
Financial deprivation (yes)	− 0.43 (0.65)	0.274	0.27 (1.31)	0.475	0.14 (1.15)	0.707	− 0.36 (0.70)	0.330	− 0.24 (0.78)	0.514
Count/adj. R^2^	351/0.14		384/0.07		353/0.08		368/0.12		361/0.06	

*Note * Only five NAV-variables that have over 40% of the “difficult”-category (excluding missing count) located at level 1 and below, have been included in the report of logistic regression analyses

Coef. = unstandardized regression slope/coefficient; OR: odds ratio

NAV3: ability to judge to what extent a health insurance covers your need of a particular health service

NAV4: ability to find out if a particular healthcare service requires a deductible

NAV5: ability to understand information on ongoing health care reforms that might affect your health care

NAV6: ability to find out about your rights as a patient or user of the health care system

NAV8: ability to find information on the quality of a particular health service

## Discussions

### Summary

The findings suggested that the HLS_19_-DIGI has acceptable psychometric properties in adolescents. A high proportion of low DHL proficiency among adolescents may represent a significant public health concern as well as a resource and capacity challenge for the healthcare services.

### Targeting and content validity

The HLS_19_-DIGI was developed based on a HL-related conceptual framework combined with a theory-based model by which the selection of items is justified. Consequently, the scale’s content validity is considered attested. However, the HLS_19_-DIGI scale obtained a relatively high positive mean person location value meaning that the person proficiencies were located at a higher level than the average difficulty of the scale. This indicates that the items could be considered to be too easy for the participants’ proficiency. Therefore, the scale could benefit from adding items that may require more challenging skills.

To access health information adolescents are expected to actively use social media and digital platforms [23, 52]. Paradoxically, item 5 that represents the ability to judge whether the information is offered with commercial interests, tends to under-discriminate even though young people are often exposed to health information [2]. Another study suggested that adolescents would prefer their family as information resources rather than social media platforms [3]. This habit could have contributed to weakening adolescents’ ability to distinguish between reliable health information and information that are provided due to commercial interests, which in turn may have caused item under-discrimination by weakening its ability to distinguish between respondents with low versus high proficiency. Previous research has demonstrated a correlation between GHL and DHL [8], and this may provide a basis for planning interventions for adolescents to develop health literacy skills enabling them to critically assess health information accessed [2].

### Overall and individual item fit

The overall chi-square statistic was statistically significant, and that could be caused by item5 with the t-value above 1.96 (t-value = 2.3). Furthermore, chi-square statistics are very sensitive to a large sample size, implying that any small differences would mostly appear statistically significant [53]. Meanwhile, a large sample size in Rasch modeling is not strictly required. A rule of thumb suggests sample size for a test of eight polytomous items with three thresholds should comprise at least 240 up to 480 persons, in which a reasonable ratio is between 10 and 20 persons for each threshold [47]. Subsequently, when the sample size was at random gradually reduced to *n* = 305 the data displayed acceptable overall data-model fit in terms of non-significant overall chi-square statistics.

In accordance with results from the Rasch analyses of the HLS_19_-DIGI when applied in adult populations [43], item5 […*to judge whether the information is offered with commercial interests*] displayed poor item fit and was the only item that under-discriminated. Item5 also displayed the highest infit MNSQ values in adults in most countries participating in the international HLS_19_-survey [43]. However, using the scale at the population level, we consider 0.7 > infit < 1.3 as sufficient [43]. As the infit was 1.16 for item5 and the T-value (2.3) was considerable higher than 1.96, this item under-discriminated and most likely measured too much of “something else” than what intended for the latent trait.

### Digital health literacy and health system navigating abilities among adolescents

Only people at level 2 and above would typically be able to understand, appraise, and apply the digital health information they access. And only people at level 1 or above would be able to use digital resources to access quality assured information. Based on the “skills” that only people at level 2 and above would typically master, we can assume that people at level 1 and below have low and inadequate DHL proficiency. In particular, people below level 1 (12%) may have problematic DHL proficiency as they probably would not be able to use digital resources to access and quality assure health information that they may need. Moreover, analyses revealed that there is a statistically significant association between DHL and adolescents’ NAV-HL. Consequently, low DHL indicates a considerable problem for properly navigating the health system and appropriately utilizing the health services available. This finding partially responds to previous research revealing that young people have less access to health care than adults [1]. There are in total 54% of the target population located at or below level 1 of DHL proficiency. This may indicate a public health concern as well as a resource and capacity challenge for the health services. The results suggest more targeted efforts, both individually and structurally, to enhance adolescents’ opportunity for better health and wellbeing.

### Strength and limitations

Novelty is the strength of the study; to our knowledge, this is the first study that we know of that has validated a unidimensional scale (HLS_19_-DIGI) measuring DHL in adolescents. A population-based study - as far as it was feasible - using nationally representative strata is another rare strength covering the population studied, holistically.

The HLS_19_-DIGI scale was psychometrically assessed in adolescents based on national data. Hence, the psychometric properties of the instrument should be further assessed using other samples or data from other countries to generalize the conclusions of the study. In addition to estimating abilities for using search engines, mastering search strategies, and critically appraising sources and selection of relevant digital health information, DHL also involves the ability to utilize digital health services, which therefore includes people’s general digital skills such as the ability to communicate via digital channels and solve technical problems entailed using digital devices. Consequently, further research on DHL should pay more attention to the latter two aspects; (1) users’ readiness to utilize digital healthcare services and (2) users’ general digital skills.

## Conclusions

The HLS_19_-DIGI scale had acceptable psychometric properties and sufficient unidimensionality offering an efficient and much needed measurement tool for use among adolescents aged 16 years and over. This is likely a useful measure in processes towards public health work. Evidence generated from this study may provide new empirical insights that are important for further adaptation of digital health information to improve adolescents’ abilities to navigate in and between health systems and health services. Apparently, DHL also involves people’s general digital skills and their ability to utilize digital health services. Therefore, further research on DHL should pay more attention to these aspects of DHL.

### Electronic supplementary material

Below is the link to the electronic supplementary material.

Supplementary Material 1

## Data Availability

The datasets used and/or analyzed during the current study are not publicly available but can be accessed by applying to the Norwegian Study Centre of HLS19 via this website: https://www.oslomet.no/forskning/forskningsprosjekter/befolkningens-helsekompetanse-hls19.
