# Supplementary material for: Associations between digital health literacy and health system navigating abilities among Norwegian adolescents: validating the HLS19-DIGI scale using Rasch modeling
Source: BMC Public Health. 2024 Jul 30;24:2043. doi: 10.1186/s12889-024-19405-w (PMC11290010; doi:10.1186/s12889-024-19405-w)
Supplement: Supplementary file 1 — Supplementary Material 1 [file 12889_2024_19405_MOESM1_ESM.docx]

**Rasch modelling**

The Rasch procedure used in this study is mainly outlined in Le, Guttersrud [1], titled: *Developing the HLS_19_-YP12 for measuring health literacy in young people: a latent trait analysis using Rasch modeling and confirmatory factor analysis*. However, the text below is more or less a transcript from the above-mentioned publication.

We tested data up against the partial credit parameterization [2] of the unidimensional Rasch model for polytomous data [3]. Models were estimated by applying the ConQuest 5 software [4], and the RUMM2030plus software [5]. For item-location estimates, RUMM2030plus uses pairwise maximum likelihood estimation (PMLE) [6], while ConQuest 5 uses marginal maximum likelihood estimation (MMLE) [7]. Normality may be considered a prerequisite when using maximum likelihood estimation. As such, the raw data obtained from the scales measuring young people’s health literacy were transformed into person-location estimates (logit values) using RUMM2030plus and ConQuest 5 software. Subsequently, the transformed data could be considered continuous and at interval level, and there is evidence of data normality when examining the normal distribution histograms. For unbiased person-location estimates, both softwares apply Warm’s mean weighted likelihood estimation (WLE) [8]. The average item-location estimate was set to 0.0 in all analyses. Using Rasch measurement theory, we evaluated dimensionality, response dependency, targeting, reliability, item fit, differential item functioning (DIF), and ordering of response categories.

**Dimensionality**

For each of the instrument versions, the dimensionality was assessed applying the combined principal component analysis (PCA) of residuals and paired *t*-test procedure [5, 9]. Based on the PCA, two subsets of items were identified. Person-location estimates on the respective two subsets were then compared using paired *t*-test. Multidimensionality is indicated when the proportion of individuals with significantly different person-location estimates on the compared subscales exceeds 5% [9, 10]. Unidimensionality is deemed to be strictly proved as opposed to multidimensionality [11]. Given a normal distribution of the differences in person-location estimates derived from the two subsets, Tennant and Pallant [12] claimed that this approach is robust enough to detect multidimensionality. In such a case, where the proportion of individuals with significantly different person-location estimates on the compared subscales exceeds 5%, we also manually performed the binomial test which is an exact test of the statistical significance of deviations from a theoretically expected distribution of observations into two categories. If the proportion lower bound 95% confidence interval in terms of number of significant t-tests is lower than or equal to .05 (5%) then the scale could be considered sufficiently unidimensional.

**Targeting of persons and items**

For a well-targeted scale, the distribution of the person-estimates should match the distribution of the item threshold estimates or difficulties [13]. As the scale is always centered on zero logits in the Rasch software, the mean person location value for a well-targeted scale would be close to the value of zero. Poor targeting may result in deflated variance in person estimates, which subsequently leads to poor person separation and deflated “test-retest” reliability indexes.

**Reliability – internal consistency**

The person separation reliability (PSR) and the person separation index (PSI) were estimated using the ConQuest 5 software and the RUMM2030plus software, respectively. In addition, Omega was estimated using the Mplus 8.6 software and the Microsoft excel-based tool to calculate ordinal Omega by standardized factor loadings and standardized residual variances [14]. Frisbie [15] has suggested that the reliability of the sum scores should exceed .85 or .65 when drawing conclusions at the individual or group level, respectively.

**Individual item fit**

Using ConQuest 5, weighted Mean Square Error (infit MNSQ) or variance-weighted fit residual was used to indicate individual item fit to the Rasch model [16]. The expected infit MNSQ value is 1, which implies perfect data-model fit. Using instruments at the population level we consider .7>infit<1.3 as sufficient [17, 18]. Furthermore, item under- and over-discrimination relative to Rasch models was indicated by values significantly different from the expected value of 1 with an absolute value of the T statistic higher than 1.96 [16, 19]. Under-discriminating items most likely measure too much of “something else” that does not correlate positively with the latent trait, with the result that they will not discriminate sufficiently well between persons with high and low standing on the latent trait [20].

A non-significant chi-square item fit statistic (p>.05) indicates good data-model fit, but the probability of detecting significant values or “misfit items” increases by the number of significance tests performed. The Bonferroni correction is one of several methods to counteract this effect [21]. For a 12-item scale, the Bonferroni adjusted chi-square probability is p/12 = .05/12=.004.

**Differential Item Functioning**

A central requirement of the Rasch model is measurement invariance, which means that items should function in the same way across different groups of people [22] such as gender and people with different health status. Items display differential item functioning (DIF) when items have different relative difficulty (uniform DIF) or discriminate differently (non-uniform DIF) for different groups of people.

We explored whether the items displayed DIF for selected person factors by two-way analysis of variance (ANOVA) of standardized residuals and inspecting graphical displays [22]. Owing to the inclusion criteria “adolescents aged 16–25 years” we dichotomized participants’ highest education level (“upper secondary school or below” versus “above upper secondary school”), and we dichotomized participants’ age accordingly (16–20 years old versus 21–25 years old). Participants’ self-reported social status on a scale from 1 to 10 was dichotomized as the two age groups probably define their level in the society based on different criteria due to life experiences, education level, living conditions and economic status. Financial deprivation was present as some reported difficulties with paying bills at the end of the month.

**Ordered response categories**

Polytomous items (here: 4-point response scale) with ordered response categories yield categorical data at the ordinal level. This implies significantly different and ordered thresholds, where thresholds are the locations at the latent trait where adjacent response categories are equally likely [22]. Disordered thresholds indicate response categories not working as intended [23].

**References**

1. Le, C., et al., *Developing the HLS_19_-YP12 for measuring health literacy in young people. A latent trait analysis using Rasch modelling and confirmatory factor analysis.* BMC Health Services Research 2022. **22**(1): p. 1485.

2. Masters, G.N., *A Rasch model for partial credit scoring.* Psychometrika, 1982. **47**(2): p. 149-174.

3. Rasch, G., *Probabilistic models for some intelligence and attainment tests*. 1980, Chicago: University of Chicago Press.

4. Adams, R., et al., *ACER ConQuest Manual*, in *ConQuest Notes and tutorials* 2022: <https://conquestmanual.acer.org/>. Accessed 17^th^ April 2022.

5. RUMM laboratory Pty Ltd., *Displaying the RUMM 2030 Analysis. Plus Edition*. 2019: RUMM laboratory Pty Ltd.

6. Katsikatsou, M., et al., *Pairwise likelihood estimation for factor analysis models with ordinal data.* Computational Statistics & Data Analysis, 2012. **56**(12): p. 4243-4258.

7. Bock, R.D. and M. Aitkin, *Marginal maximum likelihood estimation of item parameters: Application of an EM algorithm.* Psychometrika, 1981. **46**(4): p. 443-459.

8. Warm, T.A., *Weighted likelihood estimation of ability in item response theory.* Psychometrika, 1989. **54**(3): p. 427-450.

9. Smith Jr, E.V., *Understanding Rasch Measurement: Detecting and Evaluating the Impact of Multidimensionality Using Item Fit Statistics and Principal Components Analysis of Residuals.* Journal of Applied Measurement, 2002. **3**(2): p. 205-31.

10. Hagell, P., *Testing rating scale unidimensionality using the principal component analysis (PCA)/t-test protocol with the Rasch model: the primacy of theory over statistics.* Open Journal of Statistics, 2014. **4**(6): p. 456-465.

11. Strout, W.F., *A new item response theory modeling approach with applications to unidimensionality assessment and ability estimation.* Psychometrika, 1990. **55**(2): p. 293-325.

12. Tennant, A. and J.F. Pallant, *Unidimensionality Matters.* Rasch Measurement Transactions, 2006. **20**(1): p. 1048-1051.

13. Tennant, A. and P.G. Conaghan, *The Rasch measurement model in rheumatology: what is it and why use it? When should it be applied, and what should one look for in a Rasch paper?* Arthritis Care & Research, 2007. **57**(8): p. 1358-1362.

14. Dueber, D.M., *Bifactor Indices Calculator: A Microsoft Excel-based tool to calculate various indices relevant to bifactor CFA models.* 2017.

15. Frisbie, D.A., *Reliability of scores from teacher‐made tests.* Educational Measurement: Issues and Practice, 1988. **7**(1): p. 25-35.

16. Smith, R.M. *Using Item Mean Squares To Evaluate Fit to the Rasch Model*. in *The Annual Meeting of the American Educational Research Association*. 1995. San Francisco, CA.

17. Guttersrud, Ø., et al., *Rasch analyses of data collected in 17 countries: A technical report to support decision-making within the M-POHL consortium*. 2021, M-POHL: HLS_19_ Consortium.

18. Wright, B. and J.M. Linacre, *Reasonable mean-square fit values*, in *Rasch Measurement Transactions Contents*. 2022: <https://www.rasch.org/rmt/rmt83b.htm>. Accessed 22nd May 2022.

19. Adams, R.J. and M.L. Wu, *Tutorial 7 - Multidimensional models*, in *ConQuest Notes and tutorials*. 2010: <https://conquestmanual.acer.org/s2-00.html#s2-08>. Accessed 17th April 2022.

20. Masters, G.N., *Item discrimination: When more is worse.* Journal of Educational Measurement, 1988. **25**(1): p. 15-29.

21. Bland, J.M. and D.G. Altman, *Multiple significance tests: the Bonferroni method.* British Medical Journal, 1995. **310**(6973): p. 170-171.

22. Andrich, D. and I. Marais, *A Course in Rasch Measurement Theory: Measuring in the Educational, Social and Health Sciences*. 2019, Singapore: Springer.

23. Andrich, D., J. de Jong, and B. Sheridan, *Diagnostic opportunities with the Rasch model for ordered response categories.* , in *Applications of Latent Trait and Latent Class Models in the Social Sciences*, J. Rost and R. Langeheine, Editors. 1997, Waxmann Verlag GMBH: New York, NY. p. 59-70.
